# Focus On: The Use of Animal Models for the Study of Fetal Alcohol Spectrum Disorders

**Published:** 2011

**Authors:** Shannon E. Wilson, Timothy A. Cudd

**Keywords:** Fetal alcohol spectrum disorders, fetal alcohol syndrome, prenatal alcohol exposure, maternal alcohol consumption, alcohol effects, teratogenic effects of alcohol, fetal alcohol exposure research, animal models

## Abstract

Considerable efforts to educate women not to abuse alcohol during pregnancy have failed to reduce the incidence of fetal alcohol syndrome. Therefore, other approaches to limit the effects of prenatal alcohol exposure are under consideration, including the development of prevention programs and interventions. For these strategies to be as successful as possible, it also is important to improve methods for identifying affected children. The use of animal models in prenatal alcohol exposure research is critical because of the practical and ethical limitations of using human subjects for such studies. This article reviews the use of animal models in three areas of research: addressing basic questions about alcohol exposure during development; improving the identification of affected individuals; and developing approaches to reduce the impact of prenatal alcohol exposure. The various animal-model systems that have been used to study fetal alcohol spectrum disorders, each with their own specific strengths, have provided new findings that have been successfully extrapolated to human subjects, resulting in advancement of the research field and our understanding of fetal alcohol spectrum disorders.

Observational studies in humans led to the identification of the association between prenatal alcohol exposure and neurodevelopmental disorders (Lemoine et al. 1968) and were important in leading to the first description of fetal alcohol syndrome (FAS) (Jones and Smith 1973). Succeeding observational studies resulted in the identification and characterization of the comprehensive spectrum of disorders caused by prenatal alcohol exposure, now referred to as fetal alcohol spectrum disorders (FASD) (Mattson et al. 1998; Riley and McGee 2005; Sampson and Streissguth 1997). The first studies using animal models of human prenatal alcohol exposure confirmed the relationship between prenatal alcohol exposure and the disorder that was identified in human observational studies (Abel and Dintcheff 1978; Chernoff 1977; Randall et al. 1977). The paucity of autopsy reports from children with FASD, the absence of unequivocal alcohol exposure documentation, and the inability to eliminate the possibility of confounds, including environment, other substances of abuse, and nutrition, made these early animal studies both necessary and important. As a result of human observational studies and confirmative experiments in animal models of FASD, alcohol now is widely accepted by the scientific community and the public as a teratogen. Prenatal alcohol exposure is acknowledged as the leading known cause of intellectual disability in the Western world (Abel and Sokol 1986). An estimated 1 percent of live births are affected by prenatal alcohol exposure (May and Gossage 2001; O’Leary 2004; Sampson et al. 1997), and the estimated cost of FASD in the United States is $6 billion per year (Lupton et al. 2004). Although extensive efforts have been made to educate women about the dangers of drinking during pregnancy, the incidence of FAS remains essentially unchanged (Caetano et al. 2006). An estimated 120 million women in the United States drink alcohol and 10.1 percent continue to drink even after learning they are pregnant (Centers for Disease Control and Prevention 2002). Because of the high incidence and societal cost of FASD, investigators are exploring more effective ways to identify affected individuals and ways to prevent or mitigate these disorders using animal models.

Although studies in humans have been very important in identifying the spectrum of disorders, the research necessary to prevent or ameliorate the actions of prenatal alcohol exposure may be limited in human subjects because of 1) the potential for harm, 2) the limited ability to evaluate structural and functional damage, and 3) the difficulty in controlling variables in human subjects. Human studies must depend on unreliable self-reporting of alcohol consumption and have to contend with the potentially confounding effects of other drugs or tobacco that heavy drinkers often use concurrently with alcohol. In addition, controlling the variables of nutrition and genetics is difficult or impossible in human studies. Because of these limitations, investigators are performing studies in other biological platforms.

Researchers have used many different biological platforms to study different questions concerning prenatal alcohol exposure. These range in biological complexity from single cells to animal models that are very similar to humans. Cell and tissue culture experimental platforms are attractive because of their simplicity. Experimental conditions can be manipulated by controlling the tissue culture environment; therefore, the actions on the cell or cells in the culture can be directly attributed to the experimental condition. In addition, cell and tissue culture systems can allow for faster and less costly studies compared with those conducted in whole animals, which require more time, space, and materials. However, cells in culture may not behave as they do in the whole organism, and many of the disabilities caused by prenatal alcohol exposure are not characterized at the single-cell level but instead must be studied at the whole-organism level in order to appreciate the structural and/or or functional damage. The damaging effects of prenatal alcohol exposure also depend on an exposure period during development that cannot be modeled in cell culture. In addition, any identified action or effective protective action seen at the cellular level must be confirmed to be present at the more complex whole-organism level. Furthermore, preventative or ameliorative measures must be shown to cause no harm in the whole organism.

Because of these issues, animal models of FASD are used to study basic mechanistic questions and protective or ameliorative strategies and to develop better tools to screen for individuals exposed to alcohol prenatally or who show developmental damage from alcohol. However, an acceptable animal model of FASD must meet certain criteria for the results of the studies to accurately reflect what happens in humans. The first criterion is that the animal model must express a sufficiently similar functional or structural disorder in response to developmental alcohol exposure compared with humans. Animal models that meet this criterion range from those as simple as worms living in soil to nonhuman primates. The second criterion is that the mechanism of action by which the disorder is caused must be similar in humans. An example of a model that might fail this criterion would be one where the disorder under study is manifested only when alcohol exposures are so high that there is concern that the alcohol action may be mechanistically different than in humans that are exposed to far lower doses. Extensive evidence supports the conclusion that alcohol acts by different mechanisms depending on the dose, pattern of exposure, and timing of exposure relative to fetal development (Goodlett et al. 2005). If these two criteria are met, then conclusions from valid studies can potentially be rationally extrapolated to FASD in humans. This article will address more specifically the use of animal models to address three areas of research: (1) basic questions about alcohol exposure during development; (2) improving the identification of affected individuals; and (3) developing approaches to reduce the impact of prenatal alcohol exposure.

## The Use of Animal Models to Address Basic Questions About Alcohol Exposure During Development

Although many studies already have addressed how alcohol causes neurodevelopmental damage, questions remain. For example, why do differences exist in individual susceptibility to prenatal alcohol exposure? Does timing of exposure play a role in explaining the extremely heterogeneous effects? What is the role of social factors in FASD? Is there a “safe” exposure level? Does embryonic/fetal tolerance, withdrawal, or dependence play a role in developmental injuries? What are the alcohol-sensitive brain structures that are responsible for the social and other complex behavioral abnormalities? These and other complicated questions may best be addressed using animal models that are very similar to humans (i.e., translational models).

On the other hand, model systems that are very dissimilar to humans can offer unique advantages for answering mechanistic questions that cannot be addressed in humans or in translational animal models. For example, zebra fish larvae are transparent and develop external to the mother, allowing for visual study of the effects of alcohol on development (Tanguay and Riemers 2008). A simple nervous system, short generation interval, lack of a placenta, and the ability to address basic questions of development and genetics are all advantages of simple nonmammalian animal model systems such as the zebra fish, roundworm, fruit fly, and frog. Avian models also offer several advantages for developmental research. Fertile eggs are inexpensive, commercially available, and require only an incubator to develop. Researchers can open an eggshell temporarily to directly view or manipulate the embryo and easily reseal it to continue development. Significant genetic and molecular resources, such as genetic mapping, sequencing, and a database of single-nucleotide polymorphisms (http://www.ncbi.nlm.nih.gov/genome/guide/chicken/) are available, and developmental processes are very similar between avian and mammalian embryos (Smith 2008). Although the use of lower-order animal models offers opportunities to answer mechanistic questions and to test treatment approaches, verification of these findings in a more human-like or translational animal model will be important in many cases.

More advanced animals are required for the study of behavior, learning, and in cases where longer developmental periods must be studied. Rodents offer advantages that make them important models of FASD. More recently, it has been recognized that the effects of alcohol on social interactions may play an important role in FASD and may at least partially address why there is so much heterogeneity in the FASD phenotype both observationally and experimentally (reviewed by Kelly et al. 2009). Rodents offer advantages in this area of study because of their short generation interval, the ability to cross-foster, and the maternal–infant interactions in rats that effectively model some human behaviors. The sheep model offers the advantage of a long gestation, an experimental requirement for exploring repeated withdrawal during gestation as a mechanism of damage. Nonhuman primates exhibit more complex social relationships and cognitive functions and share a high degree of gene homology with humans, thus making them a good bridge between studies in other animal models, such as the rodent, and humans. For example, researchers have used nonhuman primates to study cognitive function, alcohol preference, and dopamine system function because they are so similar to humans (Kraemer et al. 2008; Roberts et al. 2004; Schneider et al. 1997, 2001, 2005). Nonhuman primates and humans also have similar gestation periods and early development. In addition, nonhuman primates’ shorter generation interval allows researchers to conduct longitudinal studies (Schneider et al. 2002). These examples illustrate how the choice of animal model must be made based on the scientific question to be studied.

## The Use of Animal Models to Improve the Identification of Affected Individuals

Early identification of FASD can result in children receiving interventions, services, and, as a consequence, improved outcomes (Streissguth et al. 1997). Therefore, early identification of children with prenatal alcohol exposure is an important goal for FASD research. Identification of FASD early in a child’s life currently is exceedingly difficult and rare; the current means are inadequate. Women who abuse alcohol during pregnancy rarely volunteer this information to health care providers. The search for better ways to identify affected individuals currently has three areas of focus. First is the identification of structural abnormalities to identify affected individuals that can be further divided into the use of facial dysmorphology and brain imaging. The second area is the identification of functional abnormalities. The third area is the identification of biomarkers different from the above methodologies.

### Facial and Brain Measurements and Imaging

Prenatal alcohol exposure can result in alterations in the development of the face, or facial dysmorphology, and accumulating experimental evidence suggests that changes in the face as a result of prenatal alcohol exposure reflect changes in brain structure and thus serve as an outward sentinel of brain damage (Sulik 2005; Sulik and Johnson 1982). Facial dysmorphology is an important screening tool for the identification of children affected by prenatal alcohol exposure. No other screening tool has yet been proved to be as specific for the identification of affected children. Currently, a diagnosis of FAS requires the identification of facial dymorphology by a highly trained specialist. This method is problematic because there are very few people who are trained to make the identification, and only a fraction of children prenatally exposed to alcohol express facial dysmorphology, at least that is currently recognizable. Efforts are underway to create camera and computer systems to detect facial dysmorphology in children (Meintjes et al. 2002, see also Wetherill and Foroud, pp. 38–41, in this issue). Researchers have used a three-dimensional facial laser to scan images from children with FAS and develop an automated diagnostic technique to identify individuals prenatally exposed to alcohol (Fang et al. 2008). The automation of the detection of facial dysmorphology also would make screening for FAS more widely available than is currently possible with the paucity of trained dysmorphologists. However, efforts to improve the sensitivity of facial dymorphology-screening tools may benefit from the use of animal models because human subjects can only be studied retrospectively. Animal subjects can be studied prospectively, providing for the possibility of identifying changes in the face caused by lower but known doses of alcohol that are more subtle than currently are recognizable by dymorphologists. This work is currently being pursued in rodent and sheep models (Anthony et al. 2010; Goodlett et al. 2010; Parnell et al. 2006).

Prenatal alcohol exposure can result in alterations in the structural development of the brain (Bookheimer and Sowell 2005; Riley et al. 2004). Recent advances in neuroimaging with the improvement of speed and resolution of magnetic resonance imaging (MRI) and functional MRI have made it possible to identify structural and functional changes in children with FASD (Spadoni et al. 2007). The use of animal models may make it possible to improve the sensitivity and specificity of neuroimaging techniques to identify FASD. Animal studies allow for control over timing and dose as well as for anatomical conformation of imaging findings. Magnetic resonance microscopy (MRM), MRI at a microscopic level of resolution of brain structures, is being used in the mouse model and provides unprecedented opportunities to define the full spectrum of alcohol’s insult to the developing brain (Parnell et al. 2009). This information will be important in confirming and extending human clinical observations. As is the case with facial dysmorphology, only with animal models can prospective studies be performed to explore structural differences below our current threshold to appreciate. Current efforts to develop brain-imaging screening tools using human subjects are limited because of the differences in alcohol-exposure dose and pattern, potential confounds of nutrition, other substances of abuse, and other environmental factors and the inability to use very high magnetic-field strengths or to corroborate findings using other histological methods.

### Functional Measures of Brain Dysfunction

Children prenatally exposed to alcohol with and without the physical features of FAS demonstrate qualitatively similar deficits in neurobehavior, including impairments in memory, attention, reaction time, visuospatial abilities, fine and gross motor skills, social and adaptive functioning, abnormal activity, reactivity, hyperactivity, attention deficits, lack of inhibition, impaired learning, reduced habituation, feeding difficulties, gait abnormalities, developmental delays, impaired motor skills, hearing abnormalities, and poor state regulation (sleep, jitteriness, and arousal abnormalities). Neurobehavioral deficits identified in children with FASD have been documented in animal studies with a variety of models (for review, see Driscoll et al. 1990; Kelly et al. 2000). Animal models have been used to identify structural damage to specific brain regions and the neural pathways that are responsible for many of these functional/behavioral deficits. Researchers now are exploiting this knowledge to develop screening tools for prenatal alcohol exposure injury by testing for specific functional deficits. Pavlovian conditioning of the eyeblink response requires a complete neural circuit that includes cerebellar Purkinje cells. Investigations using animal models have determined that the rate of acquisition of Pavlovian conditioning is diminished proportionate to cerebellar Purkinje cell loss (Brown et al. 2008; Tran et al. 2007). Cerebellar Purkinje cells are exquisitely sensitive to prenatal alcohol exposure. Eyeblink conditioning is valuable because it can be used at different stages of development and it can be used in both animals and humans, including very young children. In addition, Savage and colleagues developed a virtual Morris water-task test, in which children perform a computer task that involves locating a hidden object first by trial and error and then relocating the object by learning the relationship of the hidden object to spatial cues around the hidden object for measuring deficits in spatial navigation, providing an additional example of how findings in animal models have led to the development of functional testing in children with FASD (Hamilton et al. 2003). Animal models were used first to demonstrate that the hippocampus is important for spatial navigation, second to develop the Morris water task to measure spatial navigation in animals, third to demonstrate that loss of hippocampal function alters spatial navigation learning, and finally to establish that alcohol exposure during brain development causes hippocampal damage and impairment of spatial navigation, as measured by the Morris water task (Gianoulakis 1990; Goodlett and Peterson 1995; Morris et al. 1982; Squire 1992; Sutherland et al. 2000). It is expected that animal models will continue to play an important role in the development of additional functional testing, as in the case of the development of the virtual Morris water task, by providing the basic information on the anatomical basis of functional deficiencies and the development of learning measures that specifically measure the particular learning in question. A final important point is that functional testing may identify the more important functional abnormalities that could be present in the absence of structural deficits.

### Biomarkers

Because of the difficulty in identifying affected individuals, efforts are underway to identify biological substances in fluids or tissues whose expression changes in response to prenatal alcohol exposure or neurodevelopmental injury associated with prenatal alcohol exposure (i.e., biomarkers). Although finding effective biomarkers solely by studying human subjects may prove to be successful, using animal models poses the advantage of allowing the collection of a greater range of tissues and fluids that are sometimes necessary in the discovery stage of developing a practical screening tool. In addition, experiments using animal models can control dose and timing relative to development and pattern of alcohol exposure and can eliminate confounds (e.g., other substance use).

One approach being investigated is proteomic screening, the identification of a specific pattern of expressed proteins that is sensitive and specific to alcohol exposure. Support for this approach is based on evidence that prenatal alcohol exposure causes a specific characteristic change in the protein profile in amniotic fluid in mice (Datta et al. 2008).

A second approach is to identify specific microRNA whose expression changes (up or down) in response to prenatal alcohol exposure (Miranda et al. 2010). MicroRNAs, a recently identified family of non–protein-coding RNAs, can survive in circulation and have been found to serve as biomarkers for specific cancers (Ng et al. 2009). Efforts are underway to identify microRNA biomarkers in the sheep model of FASD (Balaraman et al. 2010). The sheep model is useful because it can be used to effectively model prenatal alcohol exposure patterns seen in women who abuse alcohol during pregnancy. Sheep have a long gestation, allowing for all drinking patterns and gestational exposures (i.e., any exposure pattern observed in humans can be tested in sheep) and the comparative equivalent of all of human prenatal brain development occurs prenatally in sheep.

A third approach is the identification of fatty acid ethyl esters (FAEEs), stable nonoxidative metabolites of alcohol metabolism that have been detected in mice, guinea pigs, rats, and sheep prenatally exposed to alcohol (Bearer et al. 1996; Brien et al. 2006; Caprara et al. 2005; Kulaga et al. 2006; Laposata et al. 2000; Littner et al. 2008; Mac et al. 1994; Soderberg et al.1999). FAEEs produced during gestation also accumulate in hair and initial infant stools in humans, and studies suggest that they may serve as an effective index of prenatal alcohol exposure (Bearer et al. 1999, 2003, 2005; Chan et al. 2003; Klein et al. 1999). Although animal models will likely serve important roles in the identification and validation of new candidate biomarkers, the possibility exists that ideal biomarkers may be expressed only in humans. Even in this case, animal model work in this area may be instrumental in the proof of concept stage of biomarker development.

## The Use of Animal Models to Develop Approaches to Reduce the Impact of Prenatal Alcohol Exposure

Animal models provide valuable tools for the development of approaches to reduce the impact of prenatal alcohol exposure. These approaches fall into three categories: (1) altering, enhancing, or enriching the social, learning, sensory, or motor environment; (2) nutritional/nutriceutical interventions; and (3) pharmacological interventions.

With increased emphasis on finding treatment strategies for FASD, researchers are investigating how the social aspects of postnatal experiences, environmental enrichment, and voluntary exercise may impact outcome in animal models of FASD. Findings from rodent studies (reviewed by Kelly et al. 2009) illustrate the potential for manipulating the postnatal social environment to ameliorate or exacerbate perinatal alcohol-induced deficits, including possible transgenerational effects. The simpler social behavior and generation interval of rodents make them useful models for determining how social context influences the effects of developmental alcohol exposure (Kelly et al. 2009). Developing a better understanding of the specific effects from varying social interactions and from environmental enrichment may have important implications for the treatment of children with FASD who consistently are characterized as having poor social skills (Kelly et al. 2009; O’Connor et al. 2006; Schonfeld et al. 2009).

Changing brain connections through new experiences (i.e., neuroplasticity) as a treatment for neurodevelopmental damage caused by prenatal alcohol exposure has shown great promise. Experiments using the rat model have demonstrated the potential therapeutic value of motor-training intervention programs that can be used with children with alcohol-related neurodevelopmental disorder. Klintsova and colleagues (2002) reported that complex motor-skills training in adult rats stimulated synapse formation in the cerebellum and that Purkinje neurons that survive an early postnatal alcohol insult are capable of substantial experience-induced plasticity.

In addition to studies using rodents, a ferret model has been developed in which alcohol exposure during a brief period of development impairs ocular dominance plasticity at a later age. This model provides a novel approach to investigate the consequences of fetal alcohol exposure and should contribute to our understanding of how alcohol disrupts neural plasticity (Medina et al. 2003). Animal studies such as these will provide a deeper understanding of the mechanisms by which prenatal alcohol exposure can disrupt the neurochemical and physiological mechanisms of synaptic plasticity that underlie cognition and learning. Information gained may lead to the development of strategies to prevent lifelong cognitive and behavioral handicaps as a result of prenatal alcohol exposure (Savage et al. 2002). The ferret model also offers a tremendous opportunity to test pharmacological treatment strategies that promote neuroplasticity (Medina et al. 2006).

A number of promising approaches to achieve neuro-protection now are under investigation, including the use of retinoids, antioxidants, neuromodulatory compounds, and nutritional/nutriceutical interventions. Research suggests that prenatal alcohol exposure disrupts retinoic acid synthesis, leading to neurodevelopmental injury and prompting studies to determine whether retinoid acid supplementation is protective (Shean and Duester 1993). Recent research has focused on oxidative stress as a potential mechanism for alcohol-induced neurodevelopmental damage. Although some rodent studies (Dong et al. 2008) have demonstrated a decrease in alcohol-induced reactive oxygen species generation in response to antioxidant therapy, other studies (Grisel and Chen 2005) using structural measures such as Purkinje cell number in the rat cerebellum or functional measures such as eyeblink classical conditioning in rats have not demonstrated a protective effect. The effectiveness of this approach remains unproven, but these studies demonstrate the strength of using multiple measures to evaluate effectiveness when testing therapeutic interventions in an animal model. The neurotrophic peptides SAL and NAP (SALLRSIPA [SAL] is an activity-dependent neurotrophic factor agonist peptide and NAPVSIPQ [NAP] is an activity-dependent neurotrophic protein agonist peptide), when given in conjunction with alcohol, have been shown to prevent neural tube deficits and the disruption of the genesis and development of serotonin (5-HT) neurons in the raphe nuclei in mice (Zhou et al. 2008). Evidence suggests that alcohol may cause neurodevelopmental injury by altering the availability of, or by increasing the requirements for, specific nutrients. Choline supplementation in the rat model administered either prenatally or perinatally has been shown to mitigate the effects of alcohol exposure in rats (Thomas 2007, 2009). Studies in pregnant sheep have demonstrated disturbances in maternal amino acid levels as a result of alcohol exposure, suggesting that alcohol mediated amino acid deficiencies may play a role in mediating neurodevelopmental damage (Ramadoss et al. 2008). The identification of nutritional protection strategies, their validation, and the establishment of safety will require the use of animal models.

## Summary

Neurodevelopmental damage as a result of prenatal alcohol exposure is a significant health problem and economic burden in the United States. Animal models have been utilized to make many of the scientific advances in the FAS/FASD field, including the validation that alcohol is a teratogen, the identification of the specific sites of injury, and the mechanisms of action. Animal models also have provided for the identification of previously unknown teratogenic effects of alcohol that were then identified in humans (Kelly et al. 2009). These include renal anomalies (DeBeukelaer et al. 1977), auditory deficits (Church 1987), spatial learning dysfunction (Blanchard et al. 1987), and impairment of eyeblink conditioning (Stanton and Goodlett 1998).

Because educational efforts have not been successful in reducing the incidence of FASD, there has been increased interest in the development of more sensitive means of identifying affected individuals and the development of prevention and treatment strategies. These efforts also will depend on the use of animal models to identify novel approaches, to validate efficacy, and to demonstrate safety. No animal develops in the same way as humans or demonstrates the intelligence and complexity of human behavior, but specific animal models accurately reflect certain aspects of human development and particular human behaviors. The correct choice of animal model involves matching the advantages of a particular animal model to the specific experimental requirements that must be met to answer a specific question. In conclusion, animal models will continue to be invaluable for improving the identification of FASD, the development of protective and treatment strategies, and for providing a greater understanding of FASD.
